# Supplementary material for: Development of a Decision Support Tool for Anticoagulation in Critically Ill Patients Admitted for SARS-CoV-2 Infection: The CALT Protocol
Source: Biomedicines. 2023 May 23;11(6):1504. doi: 10.3390/biomedicines11061504 (PMC10295063; doi:10.3390/biomedicines11061504)
Supplement: Supplementary file 1 [file biomedicines-11-01504-s001.zip › Table S1.pdf]

Table S1. Counts of Missing Data

Data	Missing data
**Weight**	3/124
**Height**	7/124
**BMI**	8/124
**Diabetes**	0/124
**Chronic Respiratory Failure**	0/124
**COPD**	0/124
**Chronic heart failure**	0/124
**Cirrhosis**	0/124
**End Stage Kidney Disease**	0/124
**Immunocompromised**	0/124
**SAPS II**	1/124
**SOFA**	1/124
**Antiplatelet drug prior to hospitalisation**	24/124
**Predominant finding on CT**	26/124
**Extension of lung injury on CT**	19/124
**Viral Variant**	21/124
**Vaccinal status**	4/124
**HFNO**	0/124
**CPAP**	0/124
**NVI**	0/124
**IV**	0/124
**ECMO**	0/124
**Prone Positioning**	24/124
**Antibiotherapy**	0/124
**Biomarkers D1**	
CRP	1/124
Leukocytes	0/124
Lymphocytes	26/124
Platelets	27/124
Fibrinogen	8/124
D-Dimers	21/124
Ferritin	24/124
LDH	48/124
PCT	24/124
Endocan	0/124
**Biomarkers D3**	
CRP	23/124
Leukocytes	17/124
Lymphocytes	60/124
Platelets	32/124
Fibrinogen	37/124
D-Dimers	43/124
Ferritin	50/124
LDH	65/124
PCT	45/124
Endocan	64/124
**Corticosteroids**	0/124
**Tocilizumab**	0/124
**Remdesivir**	1/124
**Duration of IV**	4/124
**Duration of antibiotherapy**	1/124
**ICU length of stay**	1/124
**Mortality at ICU discharge**	0/124
**Mortality at D28**	0/124

Results are presented as number (%). BMI: Body Mass Index, COPD: Chronic Obstructive Pulmonary Disease, CPAP: Continuous Positive Airway Pressure, CRP: C-reactive protein, ECMO: Extra Corporeal Membrane Oxygenation, IV: Invasive Ventilation, LDH: Lactate dehydrogenase, NIV: Non-invasive Ventilation, PCT: Procalcitonin.
