# Supplementary material for: Development of a Decision Support Tool for Anticoagulation in Critically Ill Patients Admitted for SARS-CoV-2 Infection: The CALT Protocol
Source: Biomedicines. 2023 May 23;11(6):1504. doi: 10.3390/biomedicines11061504 (PMC10295063; doi:10.3390/biomedicines11061504)
Supplement: Supplementary file 1 [file biomedicines-11-01504-s001.zip › Table S2.pdf]

Table S2. Intrinsic performance and predictive values of the CALT 1 score

**Value of CALT 1**	**Se**	**Sp**	**PPV**	**NPV**
**≥ 1**	0.96	0.45	0.28	0.98
**≥ 2**	0.83	0.69	0.37	0.95
**≥ 3**	0.79	0.72	0.39	0.94
**≥ 4**	0.5	0.96	0.74	0.9
**≥ 5**	0.33	0.96	0.65	0.87

Se: Sensibility, Sp: Specificity, PPV: Positive Predictive Value, NPV: Negative Predictive Value.
