# Supplementary material for: Development of a Decision Support Tool for Anticoagulation in Critically Ill Patients Admitted for SARS-CoV-2 Infection: The CALT Protocol
Source: Biomedicines. 2023 May 23;11(6):1504. doi: 10.3390/biomedicines11061504 (PMC10295063; doi:10.3390/biomedicines11061504)
Supplement: Supplementary file 1 [file biomedicines-11-01504-s001.zip › Table S3.pdf]

Table S3. Intrinsic performance and predictive values of the CALT 2 score

Value of CALT 2	Se	Sp	VPP	VPN
**≥ 0**	0.94	0.58	0.44	0.97
**≥ 1**	0.85	0.68	0.48	0.93
**≥ 2**	0.79	0.73	0.51	0.91
**≥ 3**	0.59	0.92	0.72	0.87
**≥ 4**	0.23	0.98	0.79	0.79
**≥ 5**	0.12	0.99	0.79	0.76
**≥ 6**	0.03	1	1	0.75

Se: Sensibility, Sp: Specificity, PPV: Positive Predictive Value, NPV: Negative Predictive Value.
